# Distinct impacts of radiological appearance on lymph node metastasis and prognosis based on solid size in clinical T1 non-small cell lung cancer

**DOI:** 10.1186/s12931-024-02727-z

**Published:** 2024-02-21

**Authors:** Zhihua Li, Cheng Pan, Wenzheng Xu, Chen Zhao, Xianglong Pan, Zhibo Wang, Weibing Wu, Liang Chen

**Affiliations:** 1 Department of Thoracic Surgery, Jiangsu Province Hospital, The First Affiliated Hospital of Nanjing Medical University, 300 Guangzhou Road, Nanjing, Jiangsu Province China; 2 Department of Thoracic Surgery, Taizhou School of Clinical Medicine, The Affiliated Taizhou People’s Hospital of Nanjing Medical University, Nanjing Medical University, Taizhou, China

**Keywords:** Non-small cell lung cancer, Radiological appearance, Lymph node metastasis, Recurrence patterns, Prognosis

## Abstract

**Background:**

Solid nodules (SN) had more aggressive features and a poorer prognosis than part-solid nodules (PSN). This study aimed to evaluate the specific impacts of nodule radiological appearance (SN vs. PSN) on lymph node metastasis and prognosis based on solid size in cT1 non-small cell lung cancer (NSCLC).

**Methods:**

Patients with cT1 NSCLC who underwent anatomical resection between 2010 and 2019 were retrospectively screened. Univariable and multivariable logistic regression analyses were adopted to evaluate the associations between nodule radiological appearance and lymph node metastasis. The log-rank test and Cox regression analyses were applied for prognostic evaluation. The cumulative recurrence risk was evaluated by the competing risk model.

**Results:**

There were 958 and 665 NSCLC patients with PSN and SN. Compared to the PSN group, the SN arm had a higher overall lymph node metastasis rate (21.7% vs. 2.7%, *P* < 0.001), including nodal metastasis at N1 stations (17.7% vs. 2.1%), N2 stations (14.0% vs. 1.6%), and skip nodal metastasis (3.9% vs. 0.6%). However, for cT1a NSCLC, no significant difference existed between SN and PSN (0 vs. 0.4%, *P* = 1). In addition, the impacts of nodule radiological appearance on lymph node metastasis varied between nodal stations. Solid NSCLC had an inferior prognosis than part-solid patients (5-year disease-free survival: 79.3% vs. 96.2%, *P* < 0.001). The survival inferiority only existed for cT1b and cT1c NSCLC, but not for cT1a. Strikingly, even for patients with nodal involvement, SN still had a poorer disease-free survival (*P* = 0.048) and a higher cumulative incidence of recurrence (*P* < 0.001) than PSN. Specifically, SN had a higher recurrence risk than PSN at each site. Nevertheless, the distribution of recurrences between SN and PSN was similar, except that N2 lymph node recurrences were more frequent in solid NSCLC (28.21% vs. 7.69%, *P* = 0.041).

**Conclusion:**

SN had higher risks of lymph node metastasis and poorer prognosis than PSN for cT1b and cT1c NSCLC, but not for cT1a. SN exhibited a greater proportion of N2 lymph node recurrence than PSN. SN and PSN needed distinct strategies for nodal evaluation and postoperative follow-up.

**Supplementary Information:**

The online version contains supplementary material available at 10.1186/s12931-024-02727-z.

## Background

According to the radiological appearance, non-small cell lung cancer (NSCLC) can be divided into three subtypes: pure ground-glass opacity (pGGO), part-solid nodules (PSN), and pure solid nodules (SN). The eighth edition of the lung cancer TNM staging system recommended the solid component size for clinical T classification [1]. Accordingly, pGGO nodules were classified as clinical Tis stage. The pGGO nodules had no risk of lymph node metastasis, and the 5-year disease-free survival (DFS) of patients with pGGO was nearly 100% [2–5]. However, for NSCLC with PSN and SN, patients with similar solid size could have a discrepant prognosis. Solid tumors exhibited more malignant behaviors, including lymph node metastasis, pleura invasion, and more high-grade histopathological subtypes, and therefore had a poorer prognosis compared to part-solid NSCLC [6, 7].

Although previous studies have provided crucial findings, the specific effects of radiological appearance (SN vs. PSN) on lymph node metastasis and recurrence patterns of cT1 NSCLC were not well demonstrated. Firstly, the detailed impacts of radiological appearance on lymph node metastasis of N1 stations, N2 stations, and skip lymph node metastasis remained unclear. Secondly, few studies suggested that the effects were distinct in NSCLC with different tumor sizes [8, 9]. For example, Kamigaichi and colleagues observed that solid NSCLC had a higher recurrence risk than part-solid NSCLC with a solid size ≤ 2 cm. However, for patients with solid size 2–3 cm, the recurrence difference did not exist [8]. Therefore, it was necessary to perform the analyses based on tumor size. Thirdly, limited study was available about the specific recurrence patterns of solid and part-solid NSCLC.

In this study, we systematically compared the risk of lymph node metastasis (including N1 stations, N2 stations, and skip lymph node metastasis) between SN and PSN based on solid tumor size (≤ 1 cm, 1–2 cm, and 2–3 cm). The univariate and multivariable logistic regression analyses were further performed to evaluate the effects of nodule radiological appearance on lymph node metastasis. Furthermore, prognostic comparison between patients with SN and PSN was conducted according to solid tumor size and pathological N stage. The univariate and multivariable Cox regression analyses were adopted for prognostic evaluation. Finally, we compared the detailed recurrence patterns of NSCLC with SN and PSN.

## Methods

### Patients screening

Patients with NSCLC who underwent anatomic surgical resection (segmentectomy and lobectomy) in our department between 2010 and 2019 were retrospectively screened. Further, patients who had a histopathological confirmed primary NSCLC and a tumor with solid component size ≤ 3 cm on high-resolution chest CT were reserved. Patients with: (1) pure GGO nodules; (2) a history of other malignancies in the last five years; (3) preoperative anti-tumor therapy; (4) adenocarcinoma in situ or minimally invasive adenocarcinoma; (5) small cell lung cancer components; (6) number of evaluated lymph node < 6 were excluded from this study [10, 11]. This study was approved by the Ethical Committee of the First Affiliated Hospital of Nanjing Medical University (2019-SR-123). Individual consent was waived for this retrospective study. The 8th edition of TNM staging classification for lung cancer was adopted in this study.

### Radiologic evaluation on thin-section CT

In this study, all patients received high-resolution chest CT (≤ 1.5 mm per section). The maximum tumor diameter and the solid component size were measured in the lung window (window width, 1500 Hounsfield units; window level, − 700 Hounsfield units). The GGO component was defined as an area with a homogenous increase in density but did not obscure the underlying vascular markings. The solid component represented an area of increased opacification that completely obscured the underlying vascular markings. Pure SN had no GGO component, while PSN were the mixture of GGO and solid components. Doctor Pan and Wang performed the measurement separately, which were further checked by Doctor Xu and Doctor Zhao. Controversies were resolved by discussion.

### Patient follow-up

In general, patients with pathological stage II-III received postoperative adjuvant therapies (radiotherapy, chemotherapy, target therapy). For those patients, physical examination, thin section chest CT, and tumor marker detection were performed every three months for the first two years. Abdominal CT (or Ultrasound B), brain MRI, and bone ECT were recommended annually (or PET-CT). For patients with p-stage I NSCLC, physical examination, thin section chest CT, and tumor markers were performed every six months during the first two years and annually thereafter. DFS was defined as the duration from the surgical date to the date of first recurrence or death from any cause.

### Statistical analysis

The Student’s t-test and the Chi-squared test (or Fisher’s exact test) were adopted for continuous and categorical variables, respectively. The univariable and multivariable logistic regression analyses were used to evaluate the associations between each variable and lymph node metastasis. Log-rank test was applied for prognostic comparison, and the Kaplan-Meier method was employed to estimate the DFS. Further, the cumulative recurrence risk was evaluated by the competing risk model. Death without tumor recurrence was considered as a competing event. Gray’s test was used for the comparison of the cumulative incidence of recurrence (CIR). The univariable and multivariable Cox regression analyses were performed to assess the impacts of various factors on NSCLC prognosis. All the analyses were performed based on R 4.1.2. The statistical significance level was set at *P* < 0.05 (two-sided).

## Results

### Incidence of lymph node metastasis in cT1 NSCLC patients with PSN and SN

**Fig. 1:**
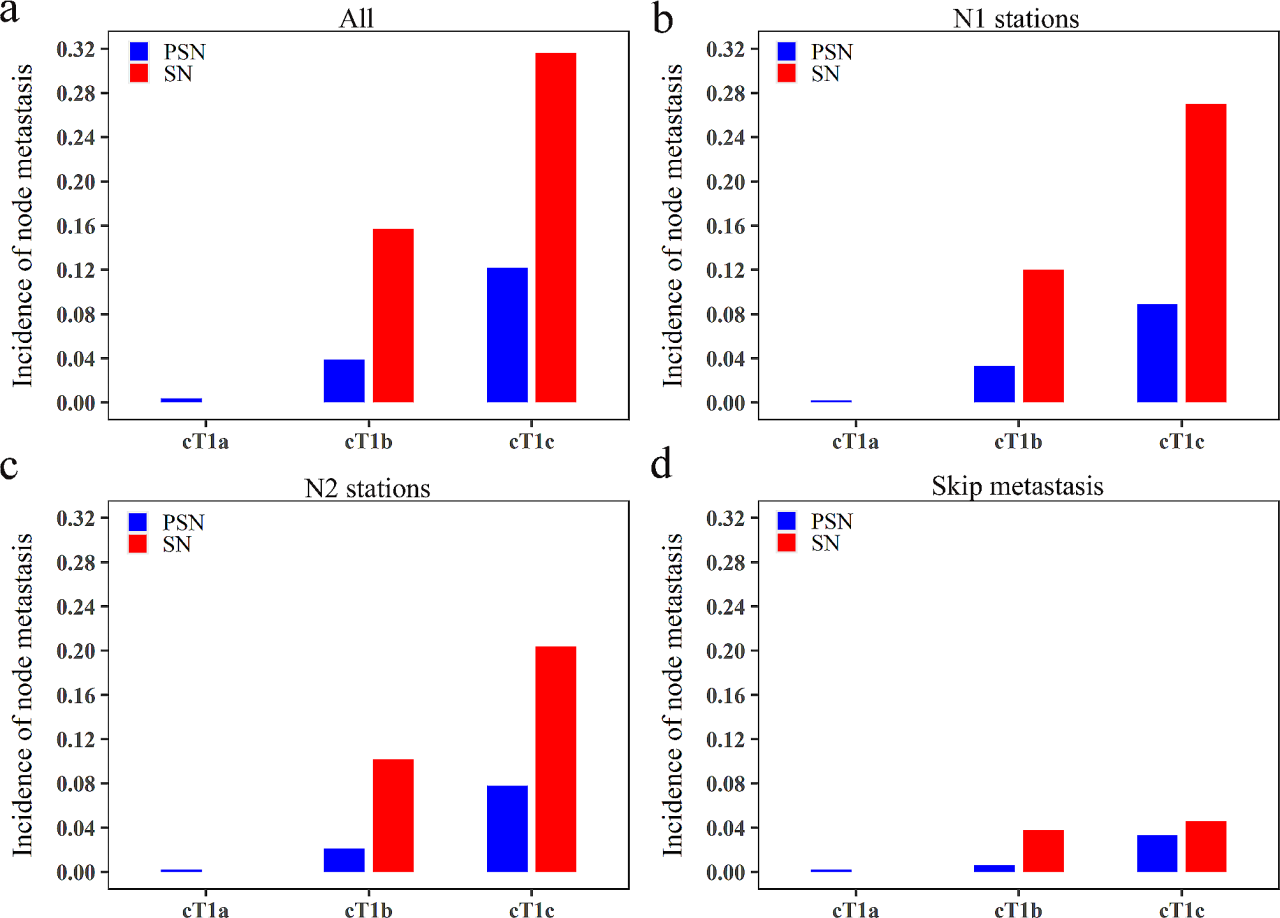
The incidence of lymph node metastasis in SN and PSN according to solid size. The incidence of overall nodal metastasis (**a**), metastasis at N1 stations (**b**), metastasis at N2 stations (**c**), and skip lymph node metastasis (**d**) in SN and PSN

There were 958 and 665 NSCLC patients with PSN and SN in this study, respectively. As shown in Table 1, patients with SN had older age, more male patients, a higher smoking rate, a higher ASA (American Society of Anesthesiologists) score, diabetes prevalence, and larger solid component size than those with PSN. The overall incidence of lymph node metastasis was 10.5% (170/1623). Compared to the PSN group, the SN arm had a higher lymph node metastasis rate (21.7% vs. 2.7%, *P* < 0.001), including N1 station (17.7% vs. 2.1%, *P* < 0.001), N2 station (14.0% vs. 1.6%, *P* < 0.001), and skip lymph node metastasis (3.9% vs. 0.6%, *P* < 0.001,). The number of metastatic lymph nodes was similar between SN and PSN (3.65 vs. 3.00, *P* = 0.230). Besides, the SN group had a higher ALK fusion rate, but a lower EGFR mutation rate compared with the PSN group. Specifically, for solid cT1a NSCLC patients, none of them had lymph node metastasis (0/37). Although two patients with PSN (2/535, 0.4%) had lymph node metastasis, the difference was not statistically significant (*P* = 1, Table 2; Fig. 1). For cT1b NSCLC, the prevalence of nodal metastasis in SN was 15.7% (54/343), 4 times higher than that in PSN (3.9%, 13/333, *P* < 0.001, Table 2). Similarly, the SN group had higher incidences of N1 (12.0% vs. 3.3%, *P* < 0.001), N2 stations (10.2% vs. 2.1%, *P* < 0.001), and skip lymph node metastasis (3.8% vs. 0.6%, *P* = 0.011) than the PSN group. For cT1c NSCLC, more than 30% (31.6%) of subjects with SN had nodal involvement, while 12.2% (*P* = 0.001) of PSN were nodal positive (Table 2). Similar findings were observed for N1 and N2 stations metastasis (Fig. 1B-C). Notably, no significant difference in skip lymph node metastasis existed between SN and PSN in cT1c NSCLC (4.6% vs. 3.3%, *P* = 0.839, Table 2; Fig. 1D).

**Table 1:** Characteristics of subjects enrolled in this study

Characteristics	PSN (*n* = 958)	SN (*n* = 665)	*P*
Age	59.44 ± 10.08	60.54 ± 10.53	0.034
Gender: female	609 (63.6%)	347 (52.2%)	< 0.001
Smoking	131 (13.7%)	156 (23.5%)	< 0.001
ASA score			
I	350 (36.5%)	220 (33.1%)	< 0.001
II	533 (55.7%)	353 (53.1%)	
III	75 (7.8%)	92 (13.8%)	
Hypertension	305 (31.8%)	226 (34.0%)	0.394
Diabetes	83 (8.7%)	89 (13.4%)	0.003
Coronary heart disease	48 (5.0%)	48 (7.2%)	0.081
Chronic respiratory diseases	34 (3.5%)	23 (3.5%)	1
Tumor location			0.001
RUL	356 (37.2%)	185 (27.8%)	
RML	75 (7.8%)	54 (8.1%)	
RLL	142 (14.8%)	117 (17.6%)	
LUL	241 (25.2%)	168 (25.3%)	
LLL	144 (15.0%)	141 (21.2%)	
Total tumor size (cm)	2.06 ± 0.88	1.95 ± 0.59	0.007
Solid size (cm)	1.06 ± 0.65	1.95 ± 0.59	< 0.001
CTR	0.51 ± 0.22	1.00 ± 0.00	< 0.001
Surgical procedure			< 0.001
Sublobar	294 (30.7%)	97 (14.6%)	
Lobar	664 (69.3%)	568 (85.4%)	
Pathological size (cm)	1.60 ± 0.71	1.84 ± 0.71	< 0.001
Histological types			< 0.001
ADC	951 (99.3%)	599 (90.1%)	
SCC	1 (0.1%)	41 (6.2%)	
Others	6 (0.6%)	25 (3.8%)	
Evaluated lymph nodes	11.41 ± 4.54	12.10 ± 4.90	0.004
Lymph node metastasis	26 (2.7%)	144 (21.7%)	< 0.001
N1 station metastasis	20 (2.1%)	118 (17.7%)	< 0.001
N2 station metastasis	15 (1.6%)	93 (14.0%)	< 0.001
Skip lymph node metastasis	6 (0.6%)	26 (3.9%)	< 0.001
Number of metastatic lymph node	3	3.65	0.230
Pleural invasion	71 (7.4%)	140 (21.1%)	< 0.001
Bronchi invasion	6 (0.6%)	44 (6.6%)	< 0.001
Solid component	53 (5.7%)	131 (21.0%)	< 0.001
Micropapillary component	38 (4.1%)	87 (13.9%)	< 0.001
Ki-67 expression level	12.86 ± 11.38	30.02 ± 23.57	< 0.001
EGFR mutation ^a^			< 0.001
Wild type	95 (21.7%)	101 (38.7%)	
Mutant type	342 (78.3%)	160 (61.3%)	
ALK fusion ^a^			
No	181 (98.9%)	96 (94.1%)	0.049
Yes	2 (1.1%)	6 (5.9%)	
ROS1 fusion ^a^			
No	181 (100.0%)	95 (97.9%)	0.232
Yes	0	2 (2.1%)	
Adjuvant therapy	107 (11.2%)	241 (36.2%)	< 0.001

PSN: part-solid nodules; SN: solid nodules; ASA: American Society of Anesthesiologists; RUL: right upper lobe; RML: right middle lobe; RLL: right lower lobe; LUL: left upper lobe; LLL: left lower lobe; CTR: consolidation to tumor ratio; ADC: adenocarcinoma; SCC: squamous cell cancer; ^a^ Genetic examination was not performed in a portion of patients

**Table 2:** Characteristics comparison of patients with PSN and SN stratified by tumor size

Characteristics	cT1a			cT1b			cT1c		
	PSN (*n* = 535)	SN (*n* = 37)	*P*	PSN (*n* = 333)	SN (*n* = 343)	*P*	PSN (*n* = 90)	SN (*n* = 285)	*P*
Age	57.76 ± 10.58	53.46 ± 11.75	0.018	61.11 ± 9.14	59.76 ± 10.16	0.069	63.27 ± 8.15	62.41 ± 10.32	0.473
Gender (female%)	358 (66.9%)	24 (64.9%)	0.940	196 (58.9%)	191 (55.7%)	0.450	55 (61.1%)	132 (46.3%)	0.020
Smoking (Ever%)	62 (11.6%)	5 (13.5%)	0.930	52 (15.6%)	74 (21.6%)	0.059	17 (18.9%)	77 (27.0%)	0.158
ASA score			0.595			0.384			0.588
I	207 (38.7%)	17 (45.9%)		117 (35.1%)	122 (35.6%)		26 (28.9%)	81 (28.4%)	
II	292 (54.6%)	17 (45.9%)		189 (56.8%)	183 (53.4%)		52 (57.8%)	153 (53.7%)	
III	36 (6.7%)	3 (8.1%)		27 (8.1%)	38 (11.1%)		12 (13.3%)	51 (17.9%)	
Hypertension	156 (29.2%)	6 (16.2%)	0.133	118 (35.4%)	116 (33.8%)	0.718	31 (34.4%)	104 (36.5%)	0.821
Diabetes	43 (8.0%)	2 (5.4%)	0.795	29 (8.7%)	43 (12.5%)	0.137	11 (12.2%)	44 (15.4%)	0.561
Coronary heart disease	22 (4.1%)	2 (5.4%)	1	19 (5.7%)	21 (6.1%)	0.947	7 (7.8%)	25 (8.8%)	0.938
Chronic respiratory diseases	18 (3.4%)	0	0.518	13 (3.9%)	12 (3.5%)	0.940	3 (3.3%)	11 (3.9%)	1
Tumor location			0.075			0.018			0.071
RUL	196 (36.6%)	15 (40.5%)		124 (37.2%)	100 (29.2%)		36 (40.0%)	70 (24.6%)	
RML	43 (8.0%)	3 (8.1%)		24 (7.2%)	27 (7.9%)		8 (8.9%)	24 (8.4%)	
RLL	76 (14.2%)	3 (8.1%)		52 (15.6%)	61 (17.8%)		14 (15.6%)	53 (18.6%)	
LUL	130 (24.3%)	4 (10.8%)		93 (27.9%)	85 (24.8%)		18 (20.0%)	79 (27.7%)	
LLL	90 (16.8%)	12 (32.4%)		40 (12.0%)	70 (20.4%)		14 (15.6%)	59 (20.7%)	
Total tumor size (cm)	1.65 ± 0.66	0.82 ± 0.16	< 0.001	2.34 ± 0.68	1.60 ± 0.28	< 0.001	3.40 ± 0.89	2.52 ± 0.28	< 0.001
Solid size (cm)	0.59 ± 0.25	0.82 ± 0.16	< 0.001	1.44 ± 0.28	1.60 ± 0.28	< 0.001	2.42 ± 0.27	2.52 ± 0.28	0.002
CTR	0.39 ± 0.18	1	< 0.001	0.65 ± 0.16	1	< 0.001	0.74 ± 0.14	1	< 0.001
Surgery (Lobectomy%)	306 (57.2%)	15 (40.5%)	0.071	273 (82.0%)	284 (82.8%)	0.859	85 (94.4%)	269 (94.4%)	1
Pathological size (cm)	1.32 ± 0.54	0.85 ± 0.30	< 0.001	1.80 ± 0.61	1.53 ± 0.49	< 0.001	2.50 ± 0.91	2.35 ± 0.61	0.087
Histological types			< 0.001			< 0.001			0.007
ADC	532 (99.4%)	34 (91.9%)		330 (99.1%)	314 (91.5%)		89 (98.9%)	251 (88.1%)	
other	3 (0.6%)	2 (5.4%)		2 (0.6%)	15 (4.4%)		1 (1.1%)	8 (2.8%)	
SCC	0	1 (2.7%)		1 (0.3%)	14 (4.1%)		0	26 (9.1%)	
Evaluated lymph nodes	10.57 ± 3.97	10.32 ± 4.12	0.716	11.82 ± 4.52	11.75 ± 4.86	0.839	14.92 ± 5.81	12.75 ± 4.96	0.001
Lymph node metastasis	2 (0.4%)	0	1	13 (3.9%)	54 (15.7%)	< 0.001	11 (12.2%)	90 (31.6%)	0.001
N1 station metastasis	1 (0.2%)	0	1	11 (3.3%)	41 (12.0%)	< 0.001	8 (8.9%)	77 (27.0%)	0.001
N2 station metastasis	1 (0.2%)	0	1	7 (2.1%)	35 (10.2%)	< 0.001	7 (7.8%)	58 (20.4%)	0.01
Skip lymph node metastasis	1 (0.2%)	0	1	2 (0.6%)	13 (3.8%)	0.011	3 (3.3%)	13 (4.6%)	0.839
Pleural invasion	23 (4.3%)	0	0.393	30 (9.0%)	56 (16.3%)	0.006	18 (20.0%)	84 (29.5%)	0.104
Bronchi invasion	1 (0.2%)	0	1	2 (0.6%)	16 (4.7%)	0.002	3 (3.3%)	28 (9.8%)	0.084
Solid component	18 (3.4%)	0	0.507	21 (6.6%)	68 (22.4%)	< 0.001	14 (15.6%)	63 (22.2%)	0.228
Micropapillary component	12 (2.3%)	1 (2.7%)	1	20 (6.2%)	41 (13.5%)	0.004	6 (6.7%)	45 (15.8%)	0.042
Ki-67 expression	10.0 (5–15)	5.0 (4.0-22.5)	0.898	10.0 (5–20)	17.0 (10-42.5)	< 0.001	17.0 (10-22.5)	30.0 (17–50)	< 0.001
EGFR mutation	179 (75.2%)	4 (36.4%)	0.012	125 (82.2%)	82 (61.7%)	< 0.001	38 (80.9%)	74 (63.2%)	0.045
ALK fusion			1			0.077			1
No	112 (98.2%)	6 (100.0%)		54 (100.0%)	52 (91.2%)		15 (100.0%)	38 (97.4%)	
Yes	2 (1.8%)	0		0	5 (8.8%)		0	1 (2.6%)	
ROS1 fusion			1			0.475			1
No	113 (100.0%)	6 (100.0%)		53 (100.0%)	51 (96.2%)		15 (100.0%)	38 (100.0%)	
Yes	0	0		0	2 (3.8%)		0	0	
Adjuvant therapy	27 (5.0%)	1 (2.7%)	0.806	38 (11.4%)	77 (22.4%)	< 0.001	42 (46.7%)	163 (57.2%)	0.104

PSN: part-solid nodules; SN: solid nodules; ASA: American Society of Anesthesiologists; RUL: right upper lobe; RML: right middle lobe; RLL: right lower lobe; LUL: left upper lobe; LLL: left lower lobe; CTR: consolidation to tumor ratio; ADC: adenocarcinoma; SCC: squamous cell cancer

**Fig. 2:**
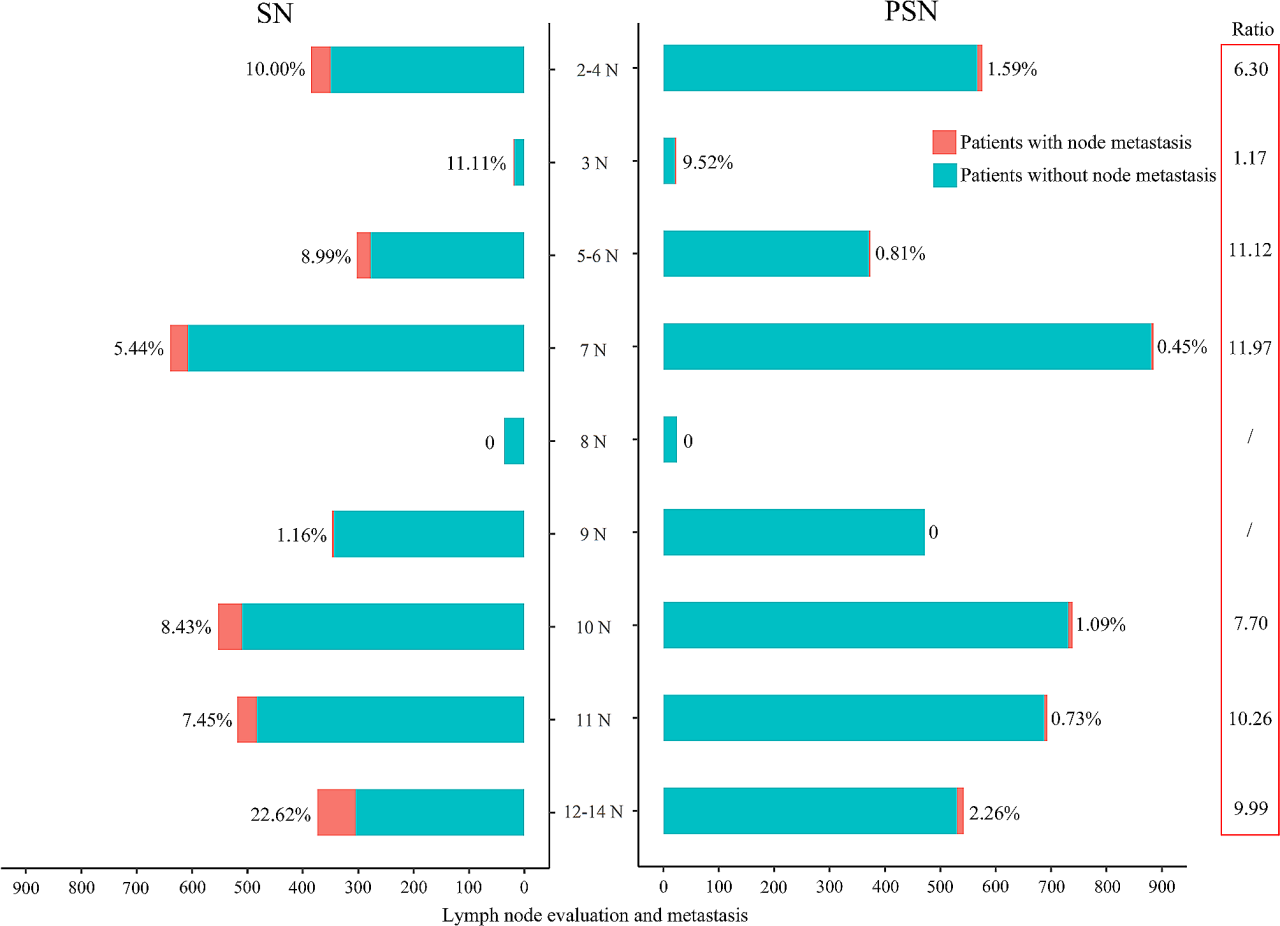
The specific patterns of lymph node evaluation and metastasis in SN and PSN at each station. Ratio = the incidence of nodal metastasis in SN/the incidence of nodal metastasis in PSN

Further, we analyzed the specific patterns of lymph node metastasis between SN and PSN (Table S1, Fig. 2). For stations 2–4, 5–6, 7, 10, 11, and 12–14, patients with SN had higher incidences of lymphatic metastasis than those with PSN (*P* < 0.05). Moreover, for stations 7 (11.97), 5–6 (11.12), and 11 (10.26), the SN group had more than ten times of lymph node metastasis risk than the PSN group. While for station 3, SN and PSN had a comparable risk of nodal involvement. No patients had station 8 metastasis, neither the SN nor PSN. Strikingly, patients with PSN had no lymphatic metastasis at station 9 (0/472), while four patients with SN had nodal metastasis (4/344, 1.16%, *P* = 0.031).

### Univariable and multivariable regression analyses

The univariable regression analysis indicated that gender, smoking, comorbidity with diabetes, solid size, nodule radiological appearance (OR = 9.91, 95%CI: 6.44–15.25, *P* < 0.001, Table 3), and number of lymph node dissection were significantly associated with lymph node metastasis. Further, the multivariable regression analyses suggested that solid size, radiological appearance (OR = 4.25, 95%CI: 2.64–6.83, *P* < 0.001), and number of lymph node dissection were independently associated with lymph node metastasis. To explore whether the effects were distinct for N1 and N2 stations, subgroup analyses were further performed. As shown in Table S2, the effects of radiological appearance on N1 and N2 lymph node metastasis were similar (N1: OR = 4.18, 95%CI: 2.46–7.11, *P* < 0.001; N2: OR = 4.25, 95%CI: 2.33–7.76, *P* < 0.001). Besides, patients with SN also had a higher risk of skip lymph node metastasis than those with PSNs (OR = 3.15, 95%CI: 1.16–8.55, *P* = 0.025, Table S3). For cT1a NSCLC, no factor was significantly associated with lymph node metastasis due to the limited samples with tumor metastasis (Table S4). For cT1b and cT1c NSCLC, the OR of SN compared to PSN was 3.58 (95%CI: 1.86–6.91, *P* < 0.001) and 3.03 (95%CI: 1.53–6.03, *P* = 0.002, Table S4), respectively. Notably, in the cT1b subgroup, comorbidity of diabetes was an independent risk factor of lymph node metastasis (OR = 2.33, 95%CI: 1.17–4.62, *P* = 0.016, Table S4).

**Table 3:** Univariable and multivariable analyses for lymph node metastasis

Characteristics	Univariate OR (95%CI)	*P*	Multivariable OR (95%CI)	*P*
Age	1.00 (0.98–1.01)	0.764		
Female vs. Male	0.60 (0.44–0.83)	0.002	0.85 (0.56–1.28)	0.432
Smoking	1.74 (1.20–2.52)	0.003	1.14 (0.70–1.86)	0.606
ASA score				
P2 vs. P1	0.80 (0.57–1.12)	0.199		
P3 vs. P1	1.04 (0.61–1.77)	0.888		
Hypertension	0.90 (0.64–1.26)	0.532		
Diabetes	1.79 (1.15–2.78)	0.009	1.30 (0.80–2.13)	0.295
Coronary heart disease	1.11 (0.58–2.13)	0.746		
Chronic respiratory diseases	1.21 (0.54–2.70)	0.651		
Tumor location		0.788		
RUL	Reference			
RLL	1.23 (0.77–1.96)			
RML	1.19 (0.65–2.18)			
LUL	0.92 (0.60–1.43)			
LLL	1.14 (0.72–1.81)			
Solid size	5.24 (4.02–6.84)	< 0.001	3.44 (2.53–4.68)	< 0.001
SN vs. PSN	9.91 (6.44–15.25)	< 0.001	4.25 (2.64–6.83)	< 0.001
Evaluated lymph nodes	1.09 (1.06–1.13)	< 0.001	1.07 (1.04–1.11)	< 0.001
Histological types		0.100		0.463
ADC	Reference		Reference	
Others	2.59 (1.10–6.10)	0.030	1.63 (0.64–4.18)	0.309
SCC	1.48 (0.61–3.56)	0.384	1.36 (0.45–2.96)	0.565
EGFR	0.86 (0.52–1.44)	0.575		
ALK fusion	1.09 (0.13–9.18)	0.934		
ROS1 fusion	0 (0-Inf)	0.999		

OR: Odds ratio; ASA: American Society of Anesthesiologists; RUL: right upper lobe; RML: right middle lobe; RLL: right lower lobe; LUL: left upper lobe; LLL: left lower lobe; PSN: part-solid nodules; SN: solid nodules; ADC: adenocarcinoma; SCC: squamous cell cancer; Inf: infinite

### Prognostic comparison of NSCLC patients with SN and PSN

**Fig. 3:**
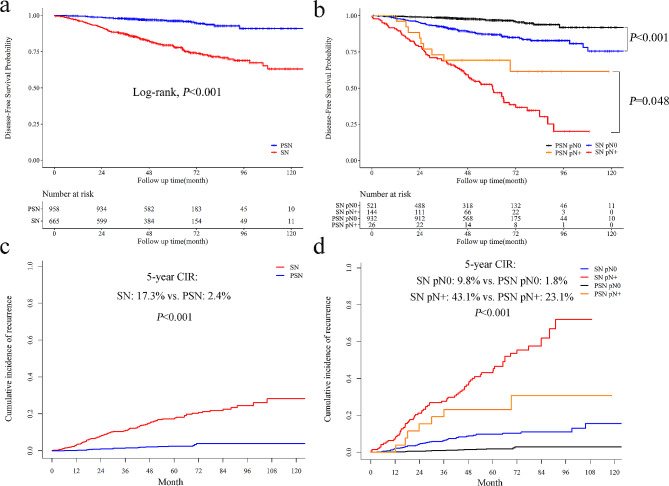
Prognostic comparison of cT1 NSCLC patients with SN and PSN. The DFS of patients with solid and part-solid cT1 NSCLC in all patients (**a**) and grouped by pathological nodal status (**b**); The CIR in all patients (**c**) with SN and PSN and grouped by pathological nodal status (**d**)

**Fig. 4:**
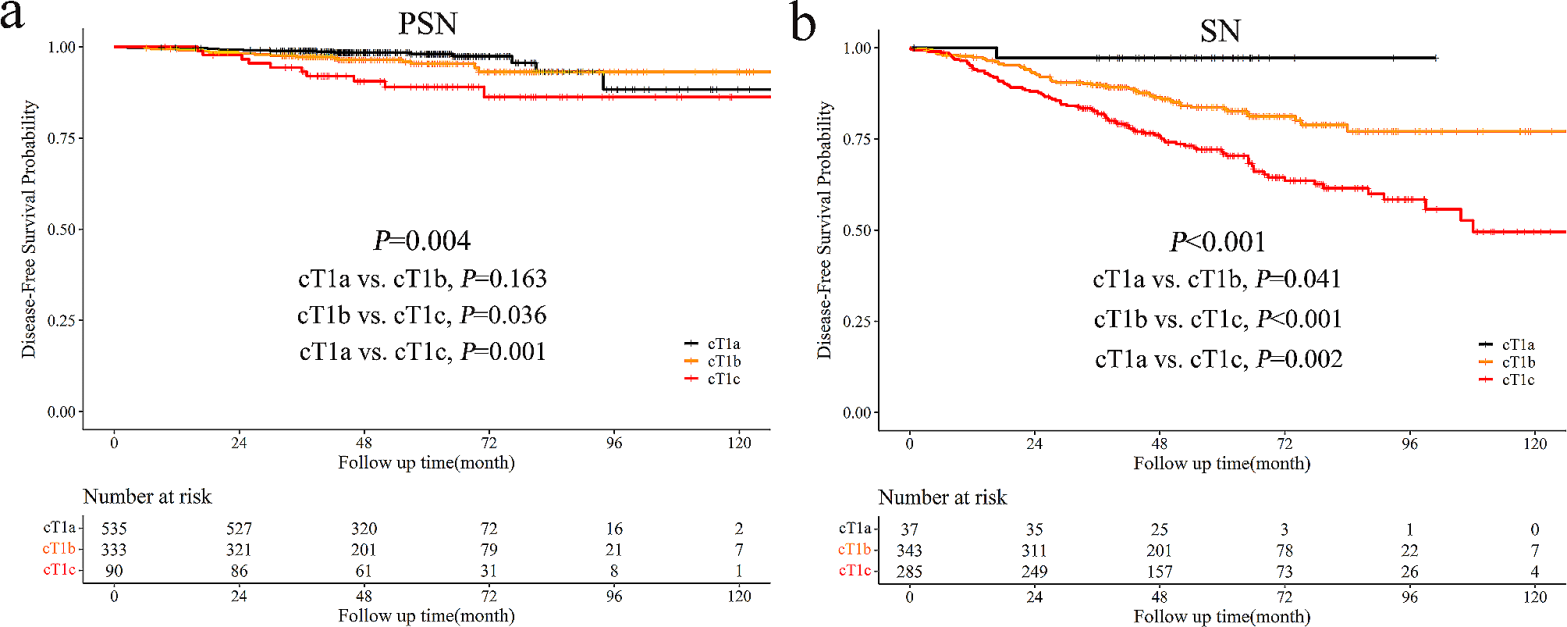
Prognostic comparison of NSCLC patients with PSN (**a**) and SN (**b**) based on solid tumor size. No significant DFS difference existed between patients with part-solid cT1a and cT1b NSCLC (*P* = 0.163). With the increase in tumor size, patients with solid cT1 NSCLC had worse prognosis

During a median follow-up of 60.1 months, a total of 187 deaths or recurrences occurred, and the 5-year DFS was 89.2% for cT1 NSCLC. Patients with SN had an inferior prognosis than those with PSN (5-year DFS: 79.3% vs. 96.2%, *P* < 0.001, Fig. 3a). For pN0 patients, the 5-year DFS were 97.0% and 86.9% for PSN and SN, respectively (*P* < 0.001). For patients with nodal involvement, solid NSCLC also had an inferior DFS than part-solid NSCLC (5-year DFS: 52.7% vs. 69.2%, *P* = 0.048, Fig. 3b). The 5-year CIR for SN was significantly higher than PSN (17.3% vs. 2.4%, *P* < 0.001, Fig. 3c). Similarly, for patients with lymphatic metastasis, the 5-year CIR for solid NSCLC was 43.1%, significantly higher than that for part-solid NSCLC (23.1%, *P* < 0.001, Fig. 3d). With the increase in solid size, the DFS of patients with SN or PSN significantly declined. However, no significant survival difference existed between part-solid cT1a and cT1b patients (*P* = 0.163, Fig. 4).

**Fig. 5:**
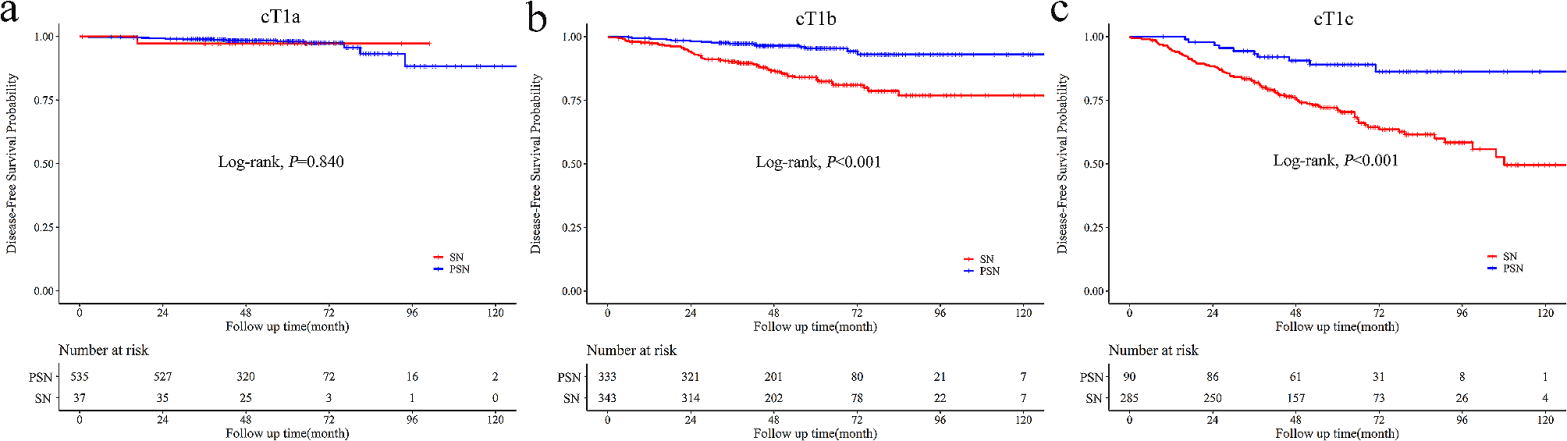
Prognostic comparison of patients with PSN and SN for cT1a (**a**), cT1b (**b**), and cT1c (**c**) NSCLC. For cT1a NSCLC, no significant DFS difference existed between patients with SN and PSN (*P* = 0.840). For cT1b and cT1c NSCLC, patients with SN had inferior prognosis than those with PSN

When stratified by tumor size, a similar prognosis was observed between cT1a patients with SN and PSN (5-year DFS: 97.2% vs. 98.1%, *P* = 0.840, Fig. 5a). For cT1b patients, SN had a poorer prognosis than PSN (5-year DFS: 84.0% vs. 95.4%, *P* < 0.001, Fig. 5b). The survival difference between patients with SN and PSN enlarged for cT1c patients (5-year DFS: 71.5% vs. 89.0%, *P* < 0.001, Fig. 5c). The multivariable regression analyses indicated that nodule radiological appearance (SN vs. PSN: HR = 2.92, 95%CI: 1.59–5.34, *P* < 0.001, Table 4) was an independent prognostic factor for cT1 NSCLC. Subgroup regression analyses in patients with nodal involvement suggested that radiological appearance (SN vs. PSN: HR = 2.03, 95%CI: 1.01–4.07, *P* = 0.046, Table S5) was the sole prognostic factor. Consistent with previous findings, radiological appearance showed no significant association with the prognosis of cT1a NSCLC (HR = 1.23, 95%CI: 0.16–9.39, *P* = 0.844, Table S6). For cT1b and cT1c NSCLC patients, nodule radiological appearance (cT1b: HR = 2.29, 95%CI: 1.26–4.13, *P* = 0.006; cT1c: HR = 2.74, 95%CI: 1.41–5.32, *P* = 0.003, Table S6) was an independent prognostic factor.

**Table 4:** Univariable and multivariable analyses for the prognosis of patients with cT1 NSCLC

Characteristics	Univariate HR (95%CI)	*P*	Multivariable HR (95%CI)	*P*
Age	1.02 (1.01–1.04)	0.002	1.03 (1.01–1.04)	0.003
Female vs. Male	0.50 (0.38–0.67)	< 0.001	0.66 (0.47–0.94)	0.020
Smoking	1.97 (1.44–2.70)	< 0.001	1.07 (0.72–1.58)	0.742
ASA score				
P2 vs. P1	1.16 (0.84–1.60)	0.355		
P3 vs. P1	1.46 (0.92–2.31)	0.109		
Hypertension	1.12 (0.83–1.50)	0.475		
Diabetes	1.10 (0.70–1.72)	0.677		
Coronary heart disease	1.10 (0.62–1.93)	0.748		
Chronic respiratory diseases	0.70 (0.29–1.70)	0.432		
SN vs. PSN	5.82 (4.07–8.31)	< 0.001	2.92 (1.59–5.34)	< 0.001
Solid size	3.02 (2.46–3.70)	< 0.001	1.25 (1.04–1.73)	0.036
Tumor location		0.395		
RUL	Reference	/		
RML	1.09 (0.62–1.93)	0.763		
RLL	1.20 (0.77–1.86)	0.422		
LUL	1.04 (0.70–1.55)	0.831		
LLL	1.48 (0.99–2.21)	0.055		
Lobectomy. vs. Sublobar	3.35 (2.03–5.52)	< 0.001	1.36 (0.80–2.31)	0.251
Histological types		0.004		0.267
ADC	Reference		Reference	/
Others	2.54 (1.30–4.98)	0.007	1.58 (0.80–3.13)	0.191
SCC	2.36 (1.28–4.34)	0.006	0.72 (0.37–1.40)	0.335
Lymph node metastasis	9.57 (7.18–12.77)	< 0.001	3.19 (2.23–4.55)	< 0.001
Evaluated lymph nodes	1.04 (1.01–1.07)	0.002	1.01 (0.98–1.03)	0.667
Pleura invasion	2.68 (1.94–3.68)	< 0.001	1.04 (0.73–1.47)	0.839
EGFR	0.77 (0.47–1.26)	0.296		
ALK fusion	1.58 (0.21–11.63)	0.655		
ROS1 fusion	0 (0-Inf)	0.997		
Adjuvant therapy	3.93 (2.15–6.32)	< 0.001	1.72 (0.85–3.01)	0.237

HR: hazard ratio; PSN: part-solid nodules; SN: solid nodules; ASA: American Society of Anesthesiologists; RUL: right upper lobe; RML: right middle lobe; RLL: right lower lobe; LUL: left upper lobe; LLL: left lower lobe; ADC: adenocarcinoma; SCC: squamous cell cancer; Inf: infinite

### Comparison of recurrence patterns between patients with PSN and SN

The detailed recurrence patterns in cT1 NSCLC patients with PSN and SN were shown in Table 5. Solid NSCLC had higher incidences of both locoregional and distant recurrences than part-solid NSCLC. Specifically, SN had higher incidences of recurrence at the ipsilateral lung, lymph node, bone, brain, contralateral lung, pleura, and liver than PSN (*P* < 0.001). Notably, solid NSCLC had a 23.62 times higher risk of N2 lymph node recurrence. Besides, 1.65% of patients with SN had recurrence at the N1 lymph nodes, while no patients with PSN had recurrence at the N1 lymph node (*P* < 0.001). Regarding the percentage of recurrence at each site, the distribution between SN and PSN was similar, except that solid NSCLC had a higher proportion of recurrence at the N2 lymph nodes (28.21% vs. 7.69, *P* < 0.041).

**Table 5:** Recurrence patterns of cT1 NSCLC with PSN and SN

	Total Number	SN			PSN			Ratio ^c^	*P*1	*P*2
		Number	Incidence ^a^	Percentage ^b^	Number	Incidence ^a^	Percentage ^b^			
**Recurrence patterns**	143	117	17.59%	100.00%	26	2.71%	100.00%	6.49	< 0.001	/
Locoregional only	18	14	2.11%	11.97%	4	0.42%	15.38%	5.02	0.003	0.743
Locoregional + distant	36	33	4.96%	28.21%	3	0.31%	11.54%	16.00	< 0.001	0.128
Distant only	83	65	9.77%	55.56%	18	1.88%	69.23%	5.20	< 0.001	0.290
Unclear	6	5	0.75%	4.27%	1	0.10%	3.85%	/	/	/
**Sites of locoregional recurrence**										
Ipsilateral lung	31	26	3.91%	22.22%	5	0.52%	19.23%	7.52	< 0.001	0.943
N1 lymph node	11	11	1.65%	9.40%	0	0	0	/	< 0.001	0.215
N2 lymph node	35	33	4.96%	28.21%	2	0.21%	7.69%	23.62	< 0.001	**0.041**
**Sites of distant recurrence**										
Bone	43	36	5.41%	30.77%	7	0.73%	26.92%	7.41	< 0.001	0.88
Brain	26	22	3.31%	18.80%	4	0.42%	15.38%	7.88	< 0.001	0.786
Contralateral lung	27	22	3.31%	18.80%	5	0.52%	19.23%	6.37	< 0.001	1
Pleura	24	20	3.01%	17.09%	4	0.42%	15.38%	7.17	< 0.001	1
Liver	12	11	1.65%	9.40%	1	0.10%	3.85%	16.50	< 0.001	0.695
Lymph node	9	7	1.05%	5.98%	2	0.21%	7.69%	5.00	0.037	0.667
Other sites	7	7	1.05%	5.98%	0	0	0	/	0.002	0.350

PSN: part-solid nodules; SN: solid nodules; ^a^: patients with recurrence/all patients with SN or PSN; ^b^: Percentage of recurrence at each site /all patients with recurrence; ^c^: Incidence in the SN group/incidence in the PSN group; *P*1: test for the incidence difference; *P*2: test for percentage difference

## Discussion

In this study, we found that solid NSCLC had higher incidences of nodal metastasis and poorer prognosis than part-solid NSCLC for cT1b and cT1c tumors, but not for cT1a. The effects were similar for metastasis of N1 stations, N2 stations, and skip nodes. However, the increased risk of nodal metastasis varied between nodal stations. Solid NSCLC had more frequent recurrences at the N2 lymph nodes.

The overall incidence of lymph node metastasis was 10.5%, similar to previous studies [12–15]. Solid NSCLC had a significantly higher lymphatic metastasis rate than part-solid NSCLC. With the increase in solid size, the prevalence of lymphatic metastasis significantly increased in both solid and part-solid NSCLC. Notably, for cT1a NSCLC, no patient with SN had lymph node metastasis, consistent with previous studies [15, 16]. However, some studies reported that 3.5-31.6% of subcentimeter lung cancer had nodal involvement [17–21]. The different measurements of tumor size and samples with distinct clinicopathologic characteristics might account for the discrepant findings. The multivariable regression analysis indicated that the risk of lymph node metastasis in solid NSCLC was three times higher than that of part-solid NSCLC when other confounding factors were adjusted, except for cT1a NSCLC. In addition, for cT1b NSCLC, the number of evaluated lymph nodes was an independent risk factor of lymph node metastasis, suggesting the necessity of adequate lymph node evaluation.

The impacts of nodule radiological appearance on lymph node metastasis at N1 station, N2 station, and skip metastasis were similar. Regarding the specific stations, we found that the SN had the highest increased risk of lymphatic metastasis at station 7, followed by 5–6, 11, 12–14, 10, and 2–4, compared to PSN. Notably, the prevalence of station 3 lymph node metastasis between SN and PSN was similar. No patient had lymph node metastasis of station 8, regardless of the SN or PSN group. Consistently, 0.7% (11/1667) of patients with c-stage I lung cancer had 8 or 9 station nodal involvement [22]. Abughararah et al. found that except for nodules in the right low lobe (1.2%), nodules in the other lobes had no station 8 lymph node metastasis [23]. Besides, the incidence of station 9 lymph node metastasis was 1.16% in the SN group, while no patient had station 9 lymph node metastasis in the PSN group. Similarly, Yazgan et al. reported that only 0.1% (1/675) of NSCLC patients had station 9 nodal metastasis [24]. In a systematic analysis of mediastinal lymph node dissection, all patients with station 9 nodal involvement had solid lung cancer. For T1 lung cancer, only one patient (1/169, 0.60%) had lymphatic metastasis at station 9 [25]. In addition, compared to mediastinal and hilar nodes, less attention was afforded to intrapulmonary lymph nodes (station 12, 13, 14) [26]. However, intrapulmonary lymph nodes had a relatively higher metastasis risk [27]. A second examination of abandoned lung samples indicated that 12% of N0 patients had metastasis of intrapulmonary lymph nodes [28]. In the present study, we found that the incidence of intrapulmonary nodal metastasis was more than 20% in solid NSCLC, but only 2.26% in part-solid NSCLC. All these findings suggested that the differences in lymph node metastasis between SN and PSN varied at various stations.

The current study found that the 5-year DFS was 96.2% and 79.3% for cT1 part-solid NSCLC and solid NSCLC, respectively. Similarly, in a study by Li and colleagues, they reported that the 5-year RFS was 96.9% for part-solid stage IA adenocarcinoma and 82.2% for solid adenocarcinoma [29]. When grouped by the solid size, the SN group had a poorer prognosis than the PSN group for cT1b and cT1c NSCLC, but not for cT1a NSCLC. Consistently, Li et al. also found that part-solid NSCLC, regardless of the solid component size, had an equivalent prognosis with solid cT1a NSCLC [29]. Conversely, Sun et al. and Hattori et al. reported that the survival inferiority still existed in solid cT1aN0M0 lung cancer [18, 21]. Lower smoking rate, younger age, and a better prognosis of patients enrolled in this study might partially account for this. In addition, few previous studies suggested that the prognostic inferiority of solid NSCLC disappeared for patients with tumors larger than 2 cm [8, 9]. However, the survival inferiority of solid NSCLC compared to part-solid NSCLC enlarged for cT1c tumors in the current study. Consistent findings were also observed by Li et al. [29]. Patients with SN had more than two times death or recurrence risk than those with PSN after adjusting for other confounding factors [30]. Notably, we observed that even for patients with lymph node metastasis, the SN group still had a poorer DFS than the PSN group. In contrast, Park et al. found that the inferior prognostic significance of SN compared to PSN was limited to those with pN0 for T1-4 NSCLC [31]. Considering the limited studies on this issue, more studies with larger sample sizes were warranted.

With regard to the specific recurrence patterns between SN and PSN, limited study was available. Recently, Park et al. observed that the solid adenocarcinoma had more frequent recurrences at ipsilateral hila, mediastinum, ipsilateral lung, and brain compared to part-solid adenocarcinoma [32]. In this study, although solid NSCLC had higher recurrence risks at each site than part-solid NSCLC, the relative percentages of recurrence at ipsilateral/contralateral lung, bone, brain, pleura, and liver between SN and PSN were similar. Strikingly, patients with N2 station lymph node recurrence were more frequent in solid NSCLC. The current study found that 1.65% and 4.96% of patients with solid cT1 NSCLC had recurrences at N1 and N2 lymph nodes. In line, Kamigaichi et al. found that 7.3% of patients with stage IA3 solid lung cancer had hilar or mediastinal nodal recurrences [33]. Hattori and colleagues also reported that for T1c solid NSCLC, 9.6% had hilar or mediastinal lymph node recurrence [34]. In addition, we found that no patients with PSN had locoregional recurrence at N1 lymph nodes. These findings supported the hypothesis that the recurrent tumors retained the characteristics of the primary tumors.

In addition to nodule radiological appearance, many other factors could also affect lymph node metastasis and NSCLC prognosis. Consistent with previous findings, tumor solid size and number of dissected lymph nodes were significantly associated with lymph node metastasis [16, 35, 36]. In the univariate logistic regression analyses, gender, smoking, and comorbidity with diabetes also showed significant associations with lymph node metastasis. However, these associations were not statistically significant anymore after adjusting for other factors [21]. Interestingly, in the cT1b subgroup, we noted that diabetes was an independent risk factor of lymph node metastasis, a finding that had not been previously reported. Shimada and colleagues found that comorbidities were significantly associated with lymph node metastasis in clinical stage IA lung cancer [37]. Nevertheless, diabetes was not analyzed separately. Therefore, further studies were necessary to demonstrate the relationship between the comorbidity of diabetes and the risk of lymph node metastasis in NSCLC. In addition, along with the nodule radiological appearance, age, gender, tumor solid size, and lymph node status were independently associated with cT1 NSCLC prognosis [21]. No significant association was observed between comorbidities and the DFS of cT1 NSCLC patients in the current study. Similarly, Seigneurin et al. reported that comorbidities were not prognostic factors for NSCLC, but for small cell lung cancer based on cases from 10 French cancer registries [38]. In contrast, a few studies have shown that comorbidities contributed to a poorer prognosis in NSCLC [39, 40]. Besides, we did not observe a significant survival benefit in patients receiving adjuvant therapy in this study. Nowadays, whether adjuvant therapy could give rise to a survival advantage for stage I NSCLC remained controversial [41, 42], which could be influenced by tumor size, histological subtypes, genetic variations and other characteristics [43–46]. More studies, especially the RCT studies, were warranted to determine the indications for adjuvant therapy in early-stage NSCLC.

Overall, the current study had several strengths. First, this study had a large sample size and could provide more credible results. Second, we excluded the pure GGO nodules. Third, systematic analyses, including logistic regression, and Cox regression analyses were performed. However, the shortages of this study should also be discussed. First, this was a single-center retrospective study, and the bias could not be fully addressed. External validation was warranted to prove our findings. Second, all the enrolled subjects were East Asians. The results could be different in Caucasian populations due to a higher smoking rate and higher proportions of squamous cell carcinoma and other histopathological subtypes. Third, molecular and genetic characteristics, as well as other radiological features, could also have substantial impacts on lymphatic metastasis and NSCLC prognosis. Fourth, although the current study had a large sample size, the sample size for solid cT1a NSCLC was limited. More studies were needed to demonstrate the differences between cT1a NSCLC shown as SN and PSN.

## Conclusions

In conclusion, solid NSCLC had higher risks of nodal metastasis and poorer prognosis than part-solid NSCLC for cT1b and cT1c tumors, but not for cT1a. Compared to part-solid NSCLC, solid NSCLC had more frequent recurrence at the N2 lymph nodes. Distinct surgical procedures and follow-up plans should be proposed for patients with solid and part-solid NSCLC.

### Electronic supplementary material

Below is the link to the electronic supplementary material.

Supplementary Material 1

## Data Availability

No datasets were generated or analysed during the current study.

## Abbreviations

ADC: Adenocarcinoma
ASA: American Society of Anesthesiologists
CI: Confidence interval
CIR: Cumulative incidence of recurrence
CTR: Consolidation to tumor ratio
DFS: Disease-free survival
HR: Hazard ratio
Inf: Infinite
LLL: Left lower lobe
LUL: Left upper lobe
NSCLC: Non-small Cell Lung Cancer
pGGO: Pure ground-glass opacity
PSN: Part-solid nodules
RLL: Right lower lobe
RML: Right middle lobe
RUL: Right upper lobe
SCC: Squamous cell cancer
SN: Solid nodules
