# Supplementary material for: Distinct impacts of radiological appearance on lymph node metastasis and prognosis based on solid size in clinical T1 non-small cell lung cancer
Source: Respir Res. 2024 Feb 21;25:96. doi: 10.1186/s12931-024-02727-z (PMC10880259; doi:10.1186/s12931-024-02727-z)
Supplement: Supplementary file 1 — Supplementary Material 1 [file 12931_2024_2727_MOESM1_ESM.docx]

**Table S1. Lymph node evaluation and metastasis at each station.**

| **Stations** | **SN** | | | **PSN** | | | **Ratios ^a^** | ***P*** |
| --- | --- | --- | --- | --- | --- | --- | --- | --- |
|  | **Dissected patients** | **Patients with metastasis** | **Incidence** | **Dissected patients** | **Patients with metastasis** | **Incidence** |  |  |
| **2-4 N** | 350 | 35 | 10.00% | 567 | 9 | 1.59% | 6.30 | <0.001 |
| **3 N** | 18 | 2 | 11.11% | 21 | 2 | 9.52% | 1.17 | 1 |
| **5-6 N** | 278 | 25 | 8.99% | 371 | 3 | 0.81% | 11.12 | <0.001 |
| **7 N** | 607 | 33 | 5.44% | 881 | 4 | 0.45% | 11.97 | <0.001 |
| **8 N** | 37 | 0 | 0 | 25 | 0 | 0 | / | 1 |
| **9 N** | 344 | 4 | 1.16% | 472 | 0 | 0 | / | 0.031 |
| **10 N** | 510 | 43 | 8.43% | 731 | 8 | 1.09% | 7.70 | <0.001 |
| **11 N** | 483 | 36 | 7.45% | 688 | 5 | 0.73% | 10.26 | <0.001 |
| **12-14 N** | 305 | 69 | 22.62% | 530 | 12 | 2.26% | 9.99 | <0.001 |

PSN, part-solid nodules; SN, solid nodules; ^a^: the incidence in SN/the incidence in PSN.

**Table S2. Univariable and multivariable analyses for lymph node metastasis of N1 and N2 stations.**

| **Characteristics** | **N1 station** | | | | **N2 station** | | | |
| --- | --- | --- | --- | --- | --- | --- | --- | --- |
|  | **Univariate OR** | ***P*** | **Multivariable OR** | ***P*** | **Univariate OR** | ***P*** | **Multivariable OR** | ***P*** |
| **Age** | 1.00 (0.98-1.02) | 0.867 | / | / | 0.99 (0.97-1.01) | 0.111 | / | / |
| **Female vs. Male** | 0.63 (0.45-0.90) | 0.010 | 0.94 (0.60-1.47) | 0.781 | 0.68 (0.46-1.00) | 0.053 | 0.98 (0.65-1.49) | 0.921 |
| **Smoking** | 1.74 (1.16-2.60) | 0.007 | 1.03 (0.61-1.74) | 0.912 | 1.44 (0.90-2.30) | 0.125 | / | / |
| **ASA score** |  |  |  |  |  |  |  |  |
| **P2 vs. P1** | 0.78 (0.54-1.14) | 0.198 |  |  | 0.70 (0.46-1.05) | 0.085 |  |  |
| **P3 vs. P1** | 1.08 (0.61-1.92) | 0.786 |  |  | 0.80 (0.41-1.59) | 0.529 |  |  |
| **Hypertension** | 1.07 (0.74-1.54) | 0.726 |  |  | 0.70 (0.45-1.10) | 0.121 |  |  |
| **Diabetes** | 1.69 (1.04-2.74) | 0.035 | 1.19 (0.70-2.03) | 0.520 | 1.90 (1.13-3.21) | 0.016 | 1.40 (0.80-2.45) | 0.236 |
| **Coronary heart disease** | 1.12 (0.55-2.28) | 0.752 |  |  | 0.76 (0.30-1.91) | 0.559 |  |  |
| **Chronic respiratory diseases** | 1.04 (0.41-2.64) | 0.941 |  |  | 0.50 (0.12-2.08) | 0.341 |  |  |
| **Tumor location** |  | 0.905 |  |  |  | 0.609 |  |  |
| **RUL** | Reference |  |  |  | Reference |  |  |  |
| **RLL** | 1.08 (0.63-1.84) |  |  |  | 1.50 (0.86-2.61) |  |  |  |
| **RML** | 1.19 (0.61-2.32) |  |  |  | 1.15 (0.54-2.48) |  |  |  |
| **LUL** | 1.02 (0.63-1.63) |  |  |  | 1.09 (0.64-1.84) |  |  |  |
| **LLL** | 1.26 (0.77-2.08) |  |  |  | 0.92 (0.49-1.69) |  |  |  |
| **Solid size** | 5.31 (3.96-7.12) | <0.001 | 3.42 (2.45-4.78) | <0.001 | 4.77 (3.48-6.54) | <0.001 | 3.10 (2.16-4.47) | <0.001 |
| **SN vs. PSN** | 10.12 (6.23-16.44) | <0.001 | 4.18 (2.46-7.11) | <0.001 | 10.22 (5.87-17.81) | <0.001 | 4.25 (2.33-7.76) | <0.001 |
| **Evaluated lymph nodes** | 1.10 (1.07-1.14) | <0.001 | 1.08 (1.04-1.12) | <0.001 | 1.07 (1.03-1.11) | <0.001 | 1.04 (1.00-1.08) | 0.049 |
| **Histological types** |  | 0.155 |  |  |  | 0.871 |  |  |
| **ADC** | Reference |  |  |  | Reference |  |  |  |
| **Others** | 2.67 (1.07-6.62) |  |  |  | 0.96 (0.23-4.07) |  |  |  |
| **SCC** | 1.17 (0.41-3.33) |  |  |  | 0.70 (0.17-2.92) |  |  |  |
| **EGFR** | 0.78 (0.45-1.34) | 0.363 |  |  | 0.95 (0.52-1.74) | 0.862 |  |  |
| **ALK fusion** | 1.27 (0.15-10.71) | 0.826 |  |  | 0 (0-Inf) | 0.991 |  |  |
| **ROS1 fusion** | 0 (0-Inf) | 0.990 |  |  | 0 (0-Inf) | 0.990 |  |  |

OR: Odds ratio; ASA, American Society of Anesthesiologists; RUL, right upper lobe; RML, right middle lobe; RLL, right lower lobe; LUL, left upper lobe; LLL, left lower lobe; PSN, part-solid nodules; SN, solid nodules; ADC, adenocarcinoma; SCC, squamous cell cancer; Inf, infinite.

**Table S3.** **Univariable and multivariable analyses for skip lymph node metastasis.**

| **Characteristics** | **Skip lymph node metastasis** | | | |
| --- | --- | --- | --- | --- |
|  | **Univariate OR (95%CI)** | ***P*** | **Multivariable OR (95%CI)** | ***P*** |
| **Age** | 0.99 (0.96-1.03) | 0.746 |  |  |
| **Female vs. Male** | 0.54 (0.26-1.09) | 0.083 | 0.70 (0.34-1.43) | 0.329 |
| **Smoking** | 1.57 (0.70-3.53) | 0.277 |  |  |
| **ASA score** |  |  |  |  |
| **P2 vs. P1** | 0.91 (0.43-1.92) | 0.804 |  |  |
| **P3 vs. P1** | 0.85 (0.24-3.05) | 0.804 |  |  |
| **Hypertension** | 0.47 (0.14-1.18) | 0.145 |  |  |
| **Diabetes** | 1.98 (0.8-4.88) | 0.137 |  |  |
| **Coronary heart disease** | 1.06 (0.25-4.51) | 0.935 |  |  |
| **Chronic respiratory diseases** | 1.86 (0.43-7.99) | 0.403 |  |  |
| **Tumor location** |  | 0.347 |  |  |
| **RUL** | Reference |  |  |  |
| **RLL** | 1.73 (0.71-4.24) |  |  |  |
| **RML** | 1.15 (0.32-4.17) |  |  |  |
| **LUL** | 0.60 (0.21-1.73) |  |  |  |
| **LLL** | 0.69 (0.22-2.17) |  |  |  |
| **Solid size** | 3.28 (1.97-5.45) | <0.001 | 2.27 (1.25-4.13) | 0.007 |
| **SN vs. PSN** | 6.46 (2.64-15.77) | <0.001 | 3.15 (1.16-8.55) | 0.025 |
| **Evaluated lymph nodes** | 1.02 (0.95-1.09) | 0.626 |  |  |
| **Histological types** |  | 0.470 |  |  |
| **ADC** | Reference |  |  |  |
| **Others** | 1.75 (0.23-13.26) |  |  |  |
| **SCC** | 2.62 (0.60-11.37) |  |  |  |
| **EGFR** | 1.77 (0.38-8.27) | 0.467 |  |  |
| **ALK fusion** | 0 (0-Inf) | 0.995 |  |  |
| **ROS1 fusion** | 1 (0-Inf) | 0.996 |  |  |

OR: Odds ratio; ASA, American Society of Anesthesiologists; RUL, right upper lobe; RML, right middle lobe; RLL, right lower lobe; LUL, left upper lobe; LLL, left lower lobe; PSN, part-solid nodules; SN, solid nodules; ADC, adenocarcinoma; SCC, squamous cell cancer; Inf, infinite.

**Table S4. Univariable and multivariable analyses for lymph node metastasis in cT1a, cT1b and cT1c NSCLC patients.**

| **Characteristics** | **cT1a** | | **cT1b** | | | **cT1c** | | |
| --- | --- | --- | --- | --- | --- | --- | --- | --- |
|  | **Univariate OR (95%CI)** | ***P*** | **Univariate OR (95%CI)** | **Multivariable OR (95%CI)** | ***P*** | **Univariate OR (95%CI)** | **Multivariable OR (95%CI)** | ***P*** |
| **Age** | 1.08 (0.93-1.26) | 0.293 | 0.98 (0.96-1.01) | / | / | 0.96 (0.94-0.99) | 0.96 (0.94-0.99) | 0.003 |
| **Female vs. Male** | 0.50 (0.03-7.97) | 0.621 | 0.40 (0.24-0.68) | 0.47 (0.25-0.87) | 0.017 | 1.29 (0.81-2.03) | / | / |
| **Smoking** | 0 (0-Inf) | 0.996 | 1.86 (1.05-3.29) | 0.93 (0.47-1.87) | 0.848 | 0.98 (0.58-1.66) | / | / |
| **ASA score** |  |  |  |  |  |  |  |  |
| **P2 vs. P1** | 411.1 (0-Inf) | 0.996 | 0.88 (0.52-1.50) |  |  | 0.48 (0.29-1.11) |  |  |
| **P3 vs. P1** | 1 (0-Inf) | 1 | 0.68 (0.25-1.85) |  |  | 0.52 (0.26-1.05) |  |  |
| **Hypertension** | 0 (0-Inf) | 0.994 | 1.06 (0.63-1.80) |  |  | 0.60 (0.36-1.03) |  |  |
| **Diabetes** | 0 (0-Inf) | 0.995 | 3.10 (1.66-5.79) | 2.33 (1.17-4.62) | 0.016 | 0.72 (0.37-1.44) |  |  |
| **Coronary heart disease** | 0 (0-Inf) | 0.995 | 1.01 (0.35-2.93) |  |  | 0.74 (0.31-1.77) |  |  |
| **Chronic respiratory diseases** | 0 (0-Inf) | 0.996 | 1.25 (0.36-4.29) |  |  | 1.09 (0.33-3.55) |  |  |
| **Tumor location** |  |  |  |  |  |  |  |  |
| **RUL** | Ref. | / | Ref. |  |  | Ref. |  |  |
| **RLL** | 0 (0-Inf) | 0.997 | 0.73 (0.35-1.51) |  |  | 1.45 (0.73-2.91) |  |  |
| **RML** | 1 (0-Inf) | 0.998 | 0.92 (0.36-2.34) |  |  | 1 (0.38-2.59) |  |  |
| **LUL** | 2 (0-Inf) | 0.997 | 0.53 (0.27-1.06) |  |  | 1.12 (0.59-2.15) |  |  |
| **LLL** | 2.08 (0.13-33.58) | 0.606 | 0.39 (0.16-0.96) |  |  | 1.78 (0.92-3.46) |  |  |
| **Solid size** | 690.3 (0.02-2147.96) | 0.173 | 9.02 (3.46-23.53) | 4.18 (1.49-11.79) | 0.007 | 2.28 (1.00-5.22) | 2.08 (0.88-4.90) | 0.094 |
| **SN vs. PSN** | 0 (0-Inf) | 0.996 | 4.60 (2.46-8.60) | 3.58 (1.86-6.91) | <0.001 | 3.31 (1.68-6.53) | 3.03 (1.53-6.03) | 0.002 |
| **Evaluated lymph nodes** | 1.12 (0.86-1.46) | 0.388 | 1.13 (1.07-1.19) | 1.10 (1.04-1.15) | <0.001 | 1.01 (0.97-1.06) | / | / |
| **Histological types** |  | 0.996 |  |  |  |  |  |  |
| **ADC** | Reference |  | Reference | / |  | Reference |  |  |
| **Others** | 0 (0-Inf) |  | 2.04 (0.57-7.3) | / | / | 2.18 (0.57-8.30) | / | / |
| **SCC** | 0 (0-Inf) |  | 0.68 (0.09-5.26) | / | / | 0.65 (0.24-1.77) | / | / |
| **EGFR** | 1276.73 (0-Inf) | 0.996 | 0.39 (0.18-1.06) |  |  | 1.91 (0.88-4.14) |  |  |
| **ALK fusion** | 0 (0-Inf) | 0.998 | 1.41 (0.15-13.41) |  |  | 0 (0-Inf) |  |  |

OR: Odds ratio; ASA, American Society of Anesthesiologists; RUL, right upper lobe; RML, right middle lobe; RLL, right lower lobe; LUL, left upper lobe; LLL, left lower lobe; PSN, part-solid nodules; SN, solid nodules; ADC, adenocarcinoma; SCC, squamous cell cancer; Inf, infinite.

**Table S5.** **Univariable and multivariable analyses for cT1 NSCLC prognosis according to pathological N stage.**

| **Characteristics** | **Pathological N+** | | **Pathological N0** | | | |
| --- | --- | --- | --- | --- | --- | --- |
|  | **Univariate** | ***P*** | **Univariate** | ***P*** | **Multivariable**  **HR (95%CI)** | ***P*** |
|  | **HR (95%CI)** |  | **HR (95%CI)** |  |  |  |
| **Age** | 1.00 (0.98-1.02) | 0.974 | 1.05 (1.03-1.07) | <0.001 | 1.04 (1.02-1.07) | <0.001 |
| **Female vs. Male** | 0.71 (0.47-1.09) | 0.126 | 0.48 (0.32-0.72) | <0.001 | 0.77 (0.48-1.25) | 0.295 |
| **Smoking** | 1.16 (0.73-1.84) | 0.536 | 2.12 (1.37-3.27) | <0.001 | 1.08 (0.63-1.86) | 0.771 |
| **ASA score** |  |  |  |  |  | 0.730 |
| **P2 vs. P1** | 0.86 (0.55-1.34) | 0.514 | 1.76 (1.09-2.85) | 0.022 | 1.22 (0.72-2.07) | 0.461 |
| **P3 vs. P1** | 0.77 (0.37-1.61) | 0.494 | 2.49 (1.33-4.66) | 0.004 | 1.28 (0.62-2.62) | 0.502 |
| **Hypertension** | 1.17 (0.75-1.82) | 0.480 | 1.18 (0.78-1.77) | 0.436 |  |  |
| **Diabetes** | 1.00 (0.57-1.74) | 0.999 | 0.66 (0.31-1.42) | 0.290 |  |  |
| **Coronary heart disease** | 1.31 (0.57-3.02) | 0.525 | 1.10 (0.51-2.37) | 0.816 |  |  |
| **Chronic respiratory diseases** | 0.53 (0.13-2.17) | 0.381 | 0.80 (0.25-2.54) | 0.709 |  |  |
| **SN vs. PSN** | 2.03 (1.02-4.07) | 0.046 | 4.20 (2.72-6.48) | <0.001 | 2.41 (1.45-4.02) | <0.001 |
| **Solid size** | 0.98 (0.65-1.47) | 0.928 | 2.73 (2.08-3.58) | <0.001 | 1.19 (0.83-1.72) | 0.347 |
| **Tumor location (Ref: RUL)** |  | 0.250 |  | 0.569 |  |  |
| **RML** | 1.68 (0.75-3.77) | 0.207 | 0.86 (0.38-1.94) | 0.708 |  |  |
| **RLL** | 1.25 (0.64-2.44) | 0.506 | 1.13 (0.63-2.03) | 0.685 |  |  |
| **LUL** | 1.91 (0.66-3.43) | 0.631 | 0.84 (0.48-1.45) | 0.522 |  |  |
| **LLL** | 1.52 (0.83-2.78) | 0.172 | 1.36 (0.79-2.33) | 0.272 |  |  |
| **Lobectomy. vs. Sublobar** | 0.64 (0.26-1.57) | 0.329 | 2.69 (1.47-4.92) | <0.001 | 1.61 (0.85-3.05) | 0.146 |
| **Histological types** |  | 0.072 |  | <0.001 |  | 0.201 |
| **ADC** | Reference | / | Reference | / | Reference |  |
| **Others** | 1.73 (0.70-4.28) | 0.235 | 2.31 (0.84-6.31) | 0.104 | 2.52 (0.91-6.96) | 0.074 |
| **SCC** | 0.22 (0.03-1.58) | 0.133 | 4.59 (2.38-8.85) | <0.001 | 1.41 (0.69-2.88) | 0.351 |
| **Evaluated lymph nodes** | 0.99 (0.95-1.03) | 0.690 | 1.03 (0.99-1.07) | 0.141 |  |  |
| **Pleura invasion** | 1.37 (0.89-2.10) | 0.154 | 1.84 (1.10-3.06) | 0.019 | 0.77 (0.45-1.33) | 0.353 |
| **EGFR** | 1.14 (0.54-2.39) | 0.730 | 0.65 (0.34-1.25) | 0.200 |  |  |
| **ALK fusion** | 0 (0-Inf) | 0.998 | 3.56 (0.46-27.43) | 0.223 |  |  |
| **Adjuvant therapy** | 1.23 (0.70-2.16) | 0.474 | 2.21 (1.37-4.45) | <0.001 | 1.67 (0.95-2.58) | 0.125 |

HR: hazard ratio; PSN, part-solid nodules; SN, solid nodules; ASA, American Society of Anesthesiologists; RUL, right upper lobe; RML, right middle lobe; RLL, right lower lobe; LUL, left upper lobe; LLL, left lower lobe; ADC, adenocarcinoma; SCC, squamous cell cancer; Inf, infinite.

**Table S6. Univariable and multivariable analyses for NSCLC prognosis in patients with cT1a, cT1b and cT1c tumors.**

| **Characteristics** | **T1a** | | **T1b** | | | **T1c** | | |
| --- | --- | --- | --- | --- | --- | --- | --- | --- |
|  | **Univariate HR (95%CI)** | ***P*** | **Univariate** **HR (95%CI)** | **Multivariable** **HR (95%CI)** | ***P*** | **Univariate** **HR (95%CI)** | **Multivariable** **HR (95%CI)** | ***P*** |
| **Age** | 1.08 (1.02-1.14) | 0.005 | 1.01 (0.99-1.04) |  |  | 1.00 (0.98-1.02) |  |  |
| **Female vs. Male** | 0.67 (0.23-1.94) | 0.464 | 0.41 (0.25-0.67) | 0.65 (0.36-1.17) | 0.153 | 0.77 (0.52-1.14) |  |  |
| **Smoking** | 1.21 (0.27-5.42) | 0.801 | 2.23 (1.35-3.66) | 1.26 (0.66-2.37) | 0.483 | 1.23 (0.80-1.90) |  |  |
| **ASA score** |  | 0.039 |  |  |  |  |  |  |
| **P2 vs. P1** | 1.98 (0.51-7.68) | 0.325 | 1.18 (0.71-1.96) |  |  | 0.85 (0.55-1.32) |  |  |
| **P3 vs. P1** | 7.53 (1.67-34.07) | 0.009 | 0.92 (0.38-2.25) |  |  | 0.76 (0.42-1.39) |  |  |
| **Hypertension** | 0.96 (0.30-3.06) | 0.942 | 0.91 (0.55-1.49) |  |  | 1.07 (0.72-1.60) |  |  |
| **Diabetes** | 2.07 (0.46-9.29) | 0.340 | 1.42 (0.73-2.77) |  |  | 0.55 (0.28-1.05) |  |  |
| **Coronary heart disease** | 1.71 (0.22-13.25) | 0.610 | 0.86 (0.31-2.37) |  |  | 0.83 (0.40-1.70) |  |  |
| **Chronic respiratory diseases** | 1.77 (0.23-13.75) | 0.583 | 0 (0-Inf) |  |  | 1.04 (0.38-2.82) |  |  |
| **SN vs. PSN** | 1.23 (0.16-9.39) | 0.844 | 3.76 (2.13-6.65) | 2.29 (1.26-4.13) | 0.006 | 3.31 (1.72-6.35) | 2.74 (1.41-5.32) | 0.003 |
| **Solid size** | 2.09 (0.25-17.38) | 0.496 | 5.22 (2.25-12.11) | 1.64 (0.67-4.00) | 0.279 | 1.50 (0.74-3.01) |  |  |
| **Location (Ref: RUL)** |  | 0.399 |  |  |  |  |  |  |
| **RML** | 0 (0-Inf) | 0.997 | 0.68 (0.26-1.76) |  |  | 1.50 (0.70-3.19) |  |  |
| **RLL** | 1.09 (0.22-5.46) | 0.917 | 0.89 (0.47-1.69) |  |  | 1.17 (0.60-2.26) |  |  |
| **LUL** | 0.47 (0.09-2.32) | 0.352 | 0.44 (0.22-0.89) |  |  | 1.49 (0.84-2.64) |  |  |
| **LLL** | 1.28 (0.36-4.59) | 0.699 | 0.75 (0.38-1.5) |  |  | 1.71 (0.96-3.06) |  |  |
| **Lobectomy. vs. Sublobar** | 1.88 (0.61-5.75) | 0.270 | 2.43 (1.05-5.62) | 0.99 (0.40-2.45) | 0.985 | 0.99 (0.43-2.26) |  |  |
| **Histological types** |  |  |  |  |  |  |  | 0.097 |
| **ADC** | Reference | / | Reference | Reference | / | Reference | Reference | / |
| **Others** | 0 (0-Inf) | 0.998 | 1.61 (0.5-5.14) | 0.91 (0.27-3.04) | 0.884 | 3.25 (1.42-7.45) | 2.27 (0.99-5.24) | 0.054 |
| **SCC** | 0 (0-Inf) | 0.999 | 4.60 (1.99-10.65) | 1.94 (0.70-5.38) | 0.206 | 0.66 (0.27-1.63) | 0.58 (0.23-1.44) | 0.239 |
| **Lymph node metastasis** | 0 (0-Inf) | 0.998 | 10.32 (6.46-16.48) | 4.49 (2.48-8.13) | <0.001 | 4.04 (2.73-5.99) | 2.64 (1.68-4.13) | <0.001 |
| **Evaluated lymph nodes** | 1.01 (0.89-1.15) | 0.867 | 1.05 (1.01-1.09) | 1.00 (0.95-1.04) | 0.835 | 0.98 (0.95-1.02) |  |  |
| **Pleura invasion** | 2.76 (0.35-21.73) | 0.334 | 1.85 (1.05-3.27) | 1.16 (0.63-2.13) | 0.629 | 1.54 (1.02-2.31) | 1.02 (0.67-1.56) | 0.914 |
| **EGFR** | 2.06 (0.25-17.12) | 0.503 | 0.35 (0.11-1.14) |  |  | 1.51 (0.72-3.20) |  |  |
| **ALK fusion** | 0 (0-Inf) | 0.999 | 2.06 (0.27-15.87) |  |  | 0 (0-Inf) |  |  |
| **Adjuvant therapy** | 2.52 (0.32-21.0) | 0.463 | 3.14 (1.85-6.80) | 1.47 (0.89-3.39) | 0.152 | 2.05 (1.95-3.76) | 1.39 (0.83-2.67) | 0.186 |

HR: hazard ratio; PSN, part-solid nodules; SN, solid nodules; ASA, American Society of Anesthesiologists; RUL, right upper lobe; RML, right middle lobe; RLL, right lower lobe; LUL, left upper lobe; LLL, left lower lobe; ADC, adenocarcinoma; SCC, squamous cell cancer; Inf, infinite.
